# Effect of folylpolyglutamate synthase A22G polymorphism on the risk and survival of patients with acute lymphoblastic leukemia

**DOI:** 10.3892/ol.2014.2175

**Published:** 2014-05-26

**Authors:** YAZMÍN GÓMEZ-GÓMEZ, JORGE ORGANISTA-NAVA, CARLOS ALBERTO RANGEL-RODRIGUEZ, BERENICE ILLADES-AGUIAR, MARÍA ELENA MORENO-GODÍNEZ, LUZ DEL CARMEN ALARCÓN-ROMERO, MARCO ANTONIO LEYVA-VÁZQUEZ

**Affiliations:** 1 Institute of Cellular Physiology, National Autonomous University of Mexico (UNAM), Mexico City 04510, Mexico; 2 Laboratory of Molecular Biomedicine, Guerrero State University, Chilpancingo, Guerrero 39090, Mexico; 3 Laboratory of Toxicology and Environmental Health, Guerrero State University, Chilpancingo, Guerrero 39090, Mexico; 4 Laboratory of Cytopathology, School of Biological Sciences, Guerrero State University, Chilpancingo, Guerrero 39090, Mexico

**Keywords:** acute lymphoblastic leukemia, folylpolyglutamate synthase, survival, A22G polymorphism

## Abstract

Folylpolyglutamate synthase (FPGS) is the key enzyme that converts the chemotherapeutic agent, methotrexate (MTX), into MTX polyglutamate. An A22G polymorphism has been found in the FPGS gene. This study aimed to evaluated whether the A22G polymorphism in the FPGS gene is associated with an increased risk of acute lymphoblastic leukemia (ALL) and whether it plays a role in increasing the survival of patients with ALL. In this study, a total of 70 patients with ALL and 100 healthy individuals were genotyped by polymerase chain reaction and sequencing methods. The homozygous variant, 22G/G [odds ratio (OR)=3.88; 95% confidence interval (CI): 2.50–6.03] and the heterozygous variant, 22A/G (OR=1.37; 95% CI: 1.26–48.95) were risk factors for ALL. Patients with the 22A/G genotype had an OR of 1.81 (95% CI: 1.57–5.74; P=0.049) and carriers of the 22G/G genotype had an OR of 2.44 (95% CI: 2.40–11.82; P=0.017) for relapse. A significant association between the A22G polymorphism and survival of patients with ALL was found (P<0.05); whereas, individuals with A/G or G/G genotypes had a decreased overall survival (log-rank test, P=0.044). Although preliminary, these data suggest that the genotypes of the A22G polymorphism may be risk factors for ALL and may play a role in the survival of patients with ALL.

## Introduction

Acute leukemias (ALs) are the most frequent type of cancer occurring in children (1). In Mexico City, ~85% of the cases were acute lymphoblastic leukemia (ALL) and 14.5% were acute myeloblastic leukemia, with a low percentage of acute biphenotypic or non-differentiated AL (1). From 1996 to 2000, a mortality rate of 63.7 per million children was recorded, which is one of the highest rates reported worldwide (2). In 2005, leukemia was the second highest cause of mortality in Guerrero in children <15 years old (3). An antineoplastic agent commonly used for the treatment of ALL is methotrexate (MTX), which was introduced to clinical oncology ~50 years ago. Folylpolyglutamate synthase (FPGS) catalyzes the polyglutamation of MTX to produce highly active metabolites (4). Certain polymorphisms at specific sites in the FPGS gene may decrease the affinity for its substrate, causing deficient polyglutamation of MTX (4). The A22G polymorphism (rs10760502), which replaces Ile with Val at position 22 of the FPGS protein, was identified in African-American, Caucasian-American, Chinese-American and Mexican-American populations (4); however, this polymorphism has not yet been studied as a factor for ALL. The present study retrospectively evaluated whether the A22G polymorphism in the FPGS gene is associated with an increased risk and survival for ALL.

## Materials and methods

### Study population

Patients (n=70) with ALL at the Pediatric Oncology Service of the State Cancer Institute ‘Arturo Beltran Ortega’ (Acapulco, Guerrero) who were diagnosed between August, 2005 and August, 2010 via bone marrow aspiration based on the French-American-British morphological criteria, cytochemical staining properties and subclassified as T- or B-lineages as previously described (5), were included in the present study. Multiagent chemotherapeutic protocols used were 96091, 96092 or CIE-10:C9.1.0 of the State Cancer Institute ‘Arturo Beltran Ortega’, as previously described (5,6). This study and the informed consent protocol were approved by the institutional review board of the Cancer Institute. Complete remission, relapse and poor outcomes were as previously defined (5,7). Risk classification was as follows: Low risk, individuals aged between one and nine years old presenting with a white blood cell (WBC) count of <50,000/mm^3^; and high risk, individuals aged less than one or more than nine years old with a WBC count of >50,000/mm^3^ (5,7). The controls included 100 healthy individuals (4–10×10^3^ leukocytes/mm^3^) without a family history of leukemia. Collectively, the subjects in the two groups were between one and 18 years old, included males and females, and were residents of Guerrero, Mexico. Patients provided written informed consent.

### Specimen collection

A bone marrow and/or blood sample was collected from the 170 participants and placed in tubes with anticoagulant. Leukocytes were purified from the whole blood sample by a selective osmotic lysis of erythrocytes; the leukocyte genomic DNA was extracted using the phenol-chloroform technique, as described previously (8).

### Genotyping

**Figure 1:**
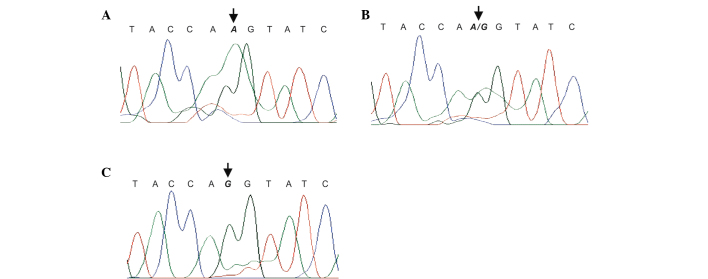
A22G polymorphism was identified by sequencing. Sequencing of the A22G polymorphism was from genomic DNA of 70 individuals with acute lymphoblastic leukemia and 100 healthy individuals without a family history of leukemia. The arrows indicate the nucleotide variants. (A) Homozygote genotype 22A/A, (B) heterozygote genotype 22A/G and (C) homozygote genotype 22G/G.

The A22G polymorphism (rs10760502) was detected by polymerase chain reaction (PCR) and sequencing using forward (5′-ACCTGCGCGCCGCTCTATTC-3′) and reverse (5′-GCTGGCCCGCCTGATACCTG-3′) primers, according to previously established protocols (9). The PCR products were sequenced using the ABI PRISM 310 Genetic analyzer (PE Applied Biosystems, Foster City, CA, USA) and sequence data were analyzed using SeqManII software (DNASTAR, Inc., Madison, WI, USA) (Fig. 1).

### Statistical analysis

Continuous data are presented as the means ± standard deviation. Categorical data were compared by the χ^2^ or Fisher’s exact tests. Univariate logistic regression analysis for the association between the risk of relapse and A22G genetic polymorphism, gender and other clinical characteristics were tested, and those factors that were significant in the univariate analysis were included in a second multivariate logistic analysis. The log-rank test and Kaplan-Meier curves were used to analyze the effects of the A22G genetic polymorphism and relapse of ALL on overall survival (OS). OS was defined as the time elapsed between the date of initial diagnosis and either death or the time of the last follow-up. The Hardy-Weinberg equilibrium (HWE) was used to determine the genetic equilibrium in the healthy group. P<0.05 was considered to indicate a statistically significant difference. All the statistical analyses were performed using SPSS software, version 21.0 (SPSS, Inc., Chicago, IL, USA) and STATA software, version 9.2 (StataCorp, College Station, TX, USA).

## Results

### Clinical characteristics

The clinical characteristics of the study population have been previously reported (5,7). Briefly, the 70 patients with ALL were aged between 1.0 and 18 years (mean age ± SD, 7.65±4.67 years), including 45 (64.29%) males and 25 (35.71%) females. Of these, 18 patients (25.71%) were aged between one and nine years old, and 52 patients (72.29%) were aged less than one year or more than nine years at the time of initial diagnosis. The median follow-up time was 38 months and the longest follow-up was six years, which occurred in only two patients. The relapse rate of patients with ALL was 68.57%.

The control group included 100 healthy individuals aged between 1.0 and 18 years old (mean ± SD, 9.99±5.49 years) with a normal leukocyte count (4–10×10^3^ leukocytes/mm^3^; median 8,000 leukocytes/mm^3^). In this group, 53 healthy individuals (53%) were male and 47 (47%) were female.

### Association of A22G polymorphism in FPGS with the risk of ALL

The genotype distribution and allele frequency of A22G polymorphism in 70 patients with ALL and 100 healthy individuals were determined. As shown in Table I, the genotype distribution of A22G polymorphism supported that expected by the HWE in healthy individuals. When the genotype frequencies were compared between the cases and controls, a statistically significant association with ALL was found (P<0.05). The homozygous variant, G/G [odds ratio (OR)=3.88; 95% confidence interval (CI): 2.50–6.03] and the heterozygote variant, A/G (OR=1.37; 95% CI: 1.26–48.95) were risk factors for ALL (Table I).

### Risk of relapse based on genotypes and other clinical characteristics

A logistic regression analysis showed that those individuals with the genotype 22A/G were 1.78-fold (95% CI: 1.56–5.63; P=0.323) more likely to relapse during treatment, while individuals with genotype 22G/G were 2.42-fold (95% CI: 1.99–11.76; P=0.272) more likely to relapse compared with individuals with genotype 22A/A (Table II). Individuals aged less than one or >10 years old with >50,000 leukocytes/mm^3^ (high risk) were 1.68-fold (95% CI: 1.36–3.72; P=0.05) more likely to have relapsed compared with individuals aged between two and nine years old with <50,000 leukocytes/mm^3^ (low risk) (Table II).

The following variables were included in the multivariate analysis: Number of leukocytes at diagnosis, age and A22G polymorphism genotypes, in order to determine whether the A22G polymorphism genotypes predicted the risk of relapse independently. Patients with the genotype 22A/G or 22G/G (OR=1.81; 95% CI: 1.57–5.74; P=0.049 and OR=2.4; 95% CI: 2.40–11.82; P=0.017, respectively), were two independent prognostic markers for the risk of relapse compared with the other variables (Table II).

### Association between A22G polymorphism and survival of patients with ALL

**Figure 2:**
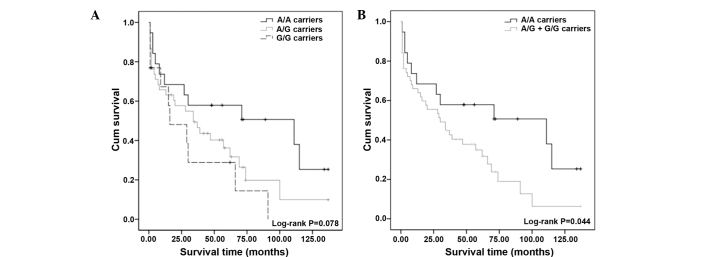
Kaplan-Meier curves of the effects of FPGS A22G polymorphism on overall survival of patients with acute lymphoblastic leukemia. (A) Association between overall survival and FPGS A22G polymorphism and (B) combined genotypes A/G + G/G vs. A/A in 70 children with ALL. Crosses indicate censoring. FPGS, folylpolyglutamate synthase.

The Kaplan-Meier survival curves showed no significant association between the FPGS A22G polymorphism and survival, although a reduction in survival after six years of follow-up among A/G and G/G carriers compared with the wild-type genotype was observed (log-rank test; P=0.078) (Fig. 2A). However, a log-rank test for the combined genotypes, 22A/G + 22G/G vs. A/A (Fig. 2B), showed a significant association between the genotype-dependent effects for the survival of patients with ALL (log-rank test; P=0.044) and increased survival was observed in those patients with genotype 22A/A (Fig. 2).

## Discussion

Agents that target the folate pathway, such as MTX, are an effective treatment for several hematologic malignancies and solid tumors. MTX is a structural analogue of folic acid that inhibits multiple enzymes in the folate pathway and requires polyglutamation by FPGS for activation. Patients with ALL have different responses to the same therapy, such as MTX resistance and subsequent relapse of ALL (10).

Leil *et al* (4) reported that the A22G polymorphism present in the FPGS gene affected individuals of Mexican-American descent and other populations conferring resistance to MTX. Additionally, Wessels *et al* (11) reported a high frequency of A22G polymorphism in patients with rheumatoid arthritis (11). Moreover, a number of studies have been conducted investigating the role of *FPGS* polymorphisms in cancer (12,13). Our data suggests that the A22G polymorphism is significantly associated with the risk of ALL (P<0.05) (Table I). However, there have been no studies evaluating the association of the A22G polymorphism with the risk of relapse and survival in patients with ALL in the literature to date.

In this study, patients with ALL predominantly showed the heterozygous A/G genotype (54.29%) of the A22G polymorphism (Table I), which is similar to the findings in the study by Wessels *et al* (11) on patients with rheumatoid arthritis (49%) (11); however, these results are in contrast to the findings of Leil *et al* (4), in African-American (15.00%), Caucasian-American (37.50%), Han Chinese-American (3.30%) and Mexican-American (32.50%) populations (4). These data suggest that the A22G polymorphism is found more frequently in patients with enzymatic activity of FPGS.

Wessels *et al* (11) and Van der Straaten *et al* (14), found no significant associations between the A22G polymorphism and MTX responses in patients with rheumatoid arthritis. Thus, whether the A22G polymorphism affects or presents a risk in specific disease therapy has not been clearly determined. However, our findings show a significant difference (P<0.05) in the frequency of A22G genotypes between children with and without relapse. Carriers of the G22G genotypes were more likely to relapse (OR=2.42; 95% CI: 1.99–11.76; P=0.272) compared with those with the AA genotype (Table II), suggesting a role for the A22G polymorphism in the risk of relapse of ALL. In the multivariate analysis, OR estimates for patients with 22G/G genotype retained their significance (OR=2.44; 95% CI: 2.40–11.82; P=0.017) in the presence of other prognostic factors, which also affected ALL outcome (age, WBC and risk classes) (Table II). A second aim of this study was to investigate the effect of the polymorphism on survival. The survival rate of G allele carriers of the A22G polymorphism was lower than that of patients carrying the A allele (Fig. 2). During follow-up, a reduction in survival among G allele carriers compared to patients with the wild-type genotype was observed (Fig. 2).

To the best of our knowledge, this study is the first to evaluate the effects of the A22G polymorphism in patients with ALL. Our data generates a novel hypotheses regarding the role of FPGS A22G polymorphism in the risk and relapse of ALL and its effects on the survival of patients with ALL. Further independent studies are required to clarify whether the associations reported in this study which just escaped statistical significance, presumably due to the limited sample size, can be corroborated. It is of interest to determine the association of FPGS variants with the folate pathway, which plays a role as an important target for anticancer therapeutics. These investigations may result in novel therapeutic regimens to correlate individual genetic variations with response to antifolate therapy and efficacy in patients with unfavorable FPGS genotypes.

## Figures and Tables

**Table I:** Genotype distribution and allele frequency of the A22G polymorphism in the FPGS gene, and association with the risk of ALL.

A22G polymorphism (rs10760502)	ALL cases (%) (n=70)	Controls (%) (n=100)	P-value	OR	95% CI	P-value	P-value HWE
Genotypes							
A/A	19 (27.14)	66 (66.00)	<0.001^a^	1.00			0.086^c^
A/G	38 (54.29)	27 (27.00)		1.37	1.26–48.95	<0.001^b^	
G/G	13 (18.57)	7 (7.00)		3.88	2.50–6.03	<0.001^b^	
A/A	19 (27.14)	66 (66.00)	<0.001^a^	1.00			
A/G+G/G	51 (72.86)	34 (34.00)		5.21	2.67–10.18	<0.001^b^	
Alleles							
A	76 (54.29)	159 (79.50)	<0.001^a^	1.00			
G	64 (45.71)	41 (20.50)		1.92	1.22–3.01	0.004^b^	

a Obtained by the χ^2^ test.

b Regression analysis, taking reference to AA genotype;

c HWE to controls.

FPGS, folylpolyglutamate synthase; ALL, acute lymphoblastic leukemia; OR, odds ratio; 95% CI, 95% confidence interval; HWE, Hardy-Weinberg equilibrium.

**Table II:** Association between A22G polymorphism in the FPGS gene and clinical characteristics with the risk of ALL recurrence.

		Univariate analysis			Multivariate analysis		
			
Characteristics	ALL cases (%)	OR	95% CI	P-value^a^	OR	95% CI	P-value^c^
Gender							
Female	25 (35.71)	1.00					
Male	45 (64.29)	1.38	0.49–3.92	0.540			
Risk at diagnosis							
Low risk	18 (25.71)	1.00					
High risk	52 (74.29)	7.64	1.90–30.73	0.004^b^	1.68	1.36–3.72	0.05^b^
A22G genotypes (rs10760502)							
A/A	19 (27.14)	1.00					
G/A	38 (54.29)	1.78	1.56–5.63	0.323	1.81	1.57–5.74	0.049^b^
G/G	13 (18.57)	2.42	1.99–11.76	0.272	2.44	2.40–11.82	0.017^b^

a P-value obtained by logistic regression analysis, taking reference to AA genotype, female, aged between two and nine years old and <50,000 leukocytes/mm^3^ (low risk).

b Significant at P<0.05.

c P-value obtained by multivariate logistic regression analysis.

FPGS, folylpolyglutamate synthase; ALL, acute lymphoblastic leukemia; OR, odds ratio; 95% CI, 95% confidence interval; low risk at diagnosis, individuals aged between one and nine years old with <50,000 leukocytes/mm^3^; high risk, individuals aged less than one and more than nine years old with >50,000 leukocytes/mm^3^.
